# Early combination of sotrovimab with nirmatrelvir/ritonavir or remdesivir is associated with low rate of persisting SARS CoV-2 infection in immunocompromised outpatients with mild-to-moderate COVID-19: a prospective single-centre study

**DOI:** 10.1080/07853890.2024.2439541

**Published:** 2024-12-11

**Authors:** I. Gentile, G. Viceconte, F. Cuccurullo, D. Pietroluongo, A. D’Agostino, M. Silvitelli, S. Mercinelli, R. Scotto, F. Grimaldi, S. Palmieri, A. Gravetti, F. Trastulli, M. Moccia, A. R. Buonomo

**Affiliations:** a Department of Clinical Medicine and Surgery, Section of Infectious Diseases, University of Naples “Federico II”, Naples, Italy; b Department of Clinical Medicine and Surgery, Hematology Unit, University of Naples “Federico II”, Naples, Italy; c Hematology Unit, “Antonio Cardarelli” Hospital, Naples, Italy; d Department of Molecular Medicine and Medical Biotechnology, University of Naples “Federico II”, Naples, Italy

**Keywords:** COVID-19, immunocompromised, combination, sotrovimab, nirmatrelvir, remdesivir

## Abstract

**Background:**

Immunocompromised patients are at high risk of developing persisting/prolonged COVID-19. Data on the early combined use of antivirals and monoclonal antibodies in this population are scarce.

**Research design and methods:**

We performed an observational, prospective study, enrolling immunocompromised outpatients with mild-to-moderate COVID-19, treated with a combination of sotrovimab plus one antiviral (remdesivir or nirmatrelvir/ritonavir) within 7 days from symptom onset. Primary outcome was hospitalization within 30 days. Secondary outcomes were: needing for oxygen therapy; development of persistent infection; death within 60 days and reinfection or relapse within 90 days.

**Results:**

We enrolled 52 patients. No patient was hospitalized within 30 days of disease onset, required oxygen administration, died within 60 days, or experienced a reinfection or clinical relapse within 90 days.

The clearance rates were 67% and 97% on the 14th day after the end of therapy and at the end of the follow-up period, respectively.

Factors associated with longer infection were initiation of therapy 3 days after symptom onset and enrollment for more than 180 days from the beginning of the study. However, only the latter factor was independently associated with a longer SARS-CoV-2 infection, suggesting a loss of efficacy of this strategy with the evolution of SARS-CoV-2 variants.

**Conclusions:**

Early administration of combination therapy with a direct antiviral and sotrovimab seems to be effective in preventing hospitalization, progression to severe COVID-19, and development of prolonged/persisting SARS-CoV-2 infection in immunocompromised patients.

## 1. Background

COVID-19 still represents a major global health problem, especially in immunocompromised patients, in whom prolonged infection and a greater risk of complications have been observed [1]. In particular, patients with impaired humoral immunity (e.g. patients with B-cell hematologic malignancies or with a depletion of B-cells) manifest a protracted course of SARS-CoV-2 infection and shed viable virus for a longer period than immunocompetent patients [1]. Moreover, these patients have an increased risk of progressing to severe COVID-19 compared with the general population [2]. A prolonged or relapsing course not only results in increased attributable morbidity or mortality but also delays chemotherapy and other therapeutic options, such as stem cell transplantation, with a negative impact on the outcome of the malignancy [3].

Early treatment with antivirals or monoclonal antibodies prevents hospitalization and severe COVID-19 in fragile patients; however, little is known about the role of such therapies in protecting highly immunosuppressed patients from developing prolonged or relapsing forms of COVID-19. Recently, some centers have described the use of a combination of monoclonal antibodies and one or two antivirals to treat COVID-19 in immunocompromised patients [4–6]. Despite this approach has shown to reach up to 80% of viral clearance in persisting infected patients, the use of combination therapy in patients that are already hospitalized with severe COVID-19, or in those who already developed persistent infection, seems to have a low impact on ‘hard’ outcomes, such as mortality or ICU-admission, especially once the persistent infection has already taken its course [4–6].

Conversely, there is a scarcity of data in the literature about the early use of combination therapy in the early phase of COVID-19 in immunocompromised outpatients. We hypothesize that the early administration of a combination therapy might prevent the development of persistent COVID-19 and, therefore, the associated morbidity and mortality. Therefore, the aim of our study was to assess the efficacy of early combination treatment with one antiviral and a monoclonal antibody, presumably active against the circulating SARS-CoV-2 variant, in non-hospitalized immunocompromised outpatients with mild-to-moderate COVID-19.

## 2. Patients and methods

### 2.1. Population

In this observational, prospective study, we consecutively enrolled immunocompromised adult patients, accessing the ambulatory service for COVID-19 outpatients of Federico II University Hospital in Naples, Southern Italy, eligible for early treatment for COVID-19 with sotrovimab and at least one antiviral between nirmatrelvir/ritonavir or remdesivir. The study period ranged from the 1st of May 1, 2023, to 30^th^ December 30, 2023. During this period, there has been a shift of SARS-CoV-2 circulating variant in Italy, from a prevalence of XBB1.5 and EG.5, towards BA2.86, and JN1, together accounting for 41% of all the infections and becoming the most prevalent at the end of December 2023 [7].

#### 2.1.1. Inclusion criteria

- Presence of at least one of the following active conditions:
  - Primary immunodeficiency.
  - Solid organ transplant on immunosuppressive therapy.
  - Chimeric antigen receptor T-cell therapy or allogeneic or autologous hematopoietic stem cell transplantation within one year.
  - Acute myeloid/lymphoblastic leukaemia within 6 months.
  - Chronic lymphoblastic leukaemia.
  - Non-Hodgkin lymphoma within 1 year from last specific therapy.
  - Plasma cellular neoplasms accompanied by hypogammaglobulinemia or receiving immunotherapy directed against B cells (bi-specific antibodies or antibody-drug conjugates against CD19, CD20, or BCMA).
  - Primary or secondary hypogammaglobulinemia.
  - Use of anti-CD20 for non-malignant conditions in the last 6 months.
- Mild-to-moderate COVID-19, with an SpO2 ≥ 94% on room air or on usual oxygen support, if already in use for chronic conditions.
- Symptoms onset ≤7 days.
- Diagnosis with SARS-CoV-2 nasopharyngeal swab (NPS) with real-time polymerase chain reaction (RT-PCR).
- Patients eligible to receive monoclonal antibody plus one antiviral agent between nirmatrelvir/ritonavir (N/r) or remdesivir.

#### 2.1.2. Exclusion criteria

- SARS CoV-2 infection during the previous 3 months.
- Age younger than 18.
- Hospitalized patients.
- Incapable of giving written informed consent
- Patients with contraindications for antiviral.
- Patients who refuse to take any of the prescribed therapies.

### 2.2. Outcomes

#### 2.2.1. Primary outcome

- Proportion of patients hospitalized for any reason within 30 days of the onset of COVID-19 symptoms

#### 2.2.2. Secondary outcomes

- Proportion of patients needing oxygen administration or with an increase in oxygen flow if on chronic oxygen therapy
- Proportion of patients with prolonged viral shedding, defined as persistence of detectable SARS-CoV-2 on NPS below 34 Ct for 14 or more days from the end of therapy.
- Time to SARS-CoV-2 clearance in days
- Death for any reason within 60 days of the onset of COVID-19 symptoms
- Reinfection or clinical relapse within 90 days of the end of therapy

### 2.3. Data collection

All patients accessing to the outpatient service for COVID-19 were screened for eligibility. Eligible patients were asked to signed written informed consent and received a single intravenous dose of Sotrovimab 500 mg and either intravenous remdesivir (200 mg on day 1 and 100 mg i.v. on days 2 and 3) or oral N/r (300/100 mg twice daily for five days). The prescribing physician chose between the two antivirals based on the patient’s history, estimated glomerular filtration rate (eGFR), and drug-drug interactions.

Patients who received remdesivir were scheduled for a second and third dose on days 2 and 3, while patients eligible for N/r received the full tablet package to complete the therapy at home. In some cases, blood samples were requested on the first visit or during follow-up to assess hepatic or renal function before or during therapy, or to measure inflammatory markers, whenever deemed appropriate for clinical reasons.

A mobile number available for 12h a day, 7 days a week, was provided to all patients to report any issues regarding the therapy or the course of the disease. All patients were followed up with weekly NPS for SARS-CoV-2 detection with RT-PCR (performed every 7 ± 3 days) until a negative result was obtained, meaning that SARS-CoV-2 was either not detectable or detectable with a cycle threshold (Ct) above 34. Patients with Ct ≥ 34 and with no symptoms for at least 3 days were considered SARS-CoV-2 negative. Isolation precautions were interrupted in these cases, and patients received formal clearance to continue immunosuppressant therapies in case they were withheld for COVID-19 by the prescribers. Patients who decided to interrupt antiviral treatment prematurely, or who were SARS-CoV-2 negative during the treatment or within 3 days from the end of the therapy, were excluded from the study.

After reaching viral clearance, patients were instructed to inform the clinical center in case of any COVID-19 symptoms or positive SARS-CoV-2 result on NPS performed for any reason. In these cases, patients received an appointment for clinical examination and repeated SARS-CoV-2 testing using RT-PCR. In March 2024, every patient included in the observation period received a call from the investigators to specifically assess if they were still alive and if they had any reinfection or relapse. Data were crossed with a regional digital platform, in which positive SARS-CoV-2 samples were recorded from general practitioners, hospitals, laboratories, and pharmacies.

### 2.4. Definitions

Prolonged SARS-CoV-2 infection was defined as the persistence of detectable SARS-CoV-2 on NPS below 34 Ct for 14 or more days from the end of therapy.

Monoclonal Antibody Screening Score (MASS) assigned a score to each of the original US FDA EUA criteria (released in November 2020) as follows: age ≥ 65 y (2), BMI ≥ 35 kg/m2 (2), diabetes mellitus (2), chronic kidney disease (3), cardiovascular disease in a patient 55 y and older (2), chronic respiratory disease in a patient 55 y and older (3), hypertension in a patient 55 y and older (1), and immunocompromised status (3). The maximum score is 18 [8,9].

### 2.5. Statistical analysis

Statistical analyses were performed using IBM SPSS Statistics for Windows version 27 (SPSS Inc. Chicago, IL, USA). Continuous variables are reported as median and interquartile range, and categorical variables as frequencies and percentages. Categorical variables were compared using the chi-squared test and Fisher’s exact test when appropriate. Continuous variables were compared using Student’s t-test (parametric variables) or the Mann–Whitney U-test for nonparametric variables. Kaplan–Meier analysis was performed to estimate the cumulative percentage of viral clearance over time among patients treated within 180 days and those treated later than 180 days from study onset. A Kaplan-Meier analysis was also performed to estimate the cumulative percentage of viral clearance over time among patients who started treatment for SARS-CoV-2 within 3 days from the onset of symptoms and those treated later than 3 days from symptom onset. Subsequently, a Cox Regression analysis was performed to calculate the risk factors for prolonged SARS-CoV-2 infection (> 14 days). All covariates that were significantly associated with the dependent variable, or those with a p-value <0.2, were subsequently inserted into a multi-variate Cox regression model to calculate the adjusted hazard ratio (aHR). The confidence interval was set at 95% for the interpretation of the results. A significance level of 0.05 was set to interpret the results.

## 3. Results

During the study period (from the 1st of May 1, 2023 to 30^th^ December 30, 2023), 253 patients were screened for inclusion criteria, and 52 were considered eligible. Of the 201 non-eligible patients, 184/201 (91%) had one or more underlying conditions different from those listed in the inclusion criteria, seven (3.4%) needed for hospital admission at the presentation and 10/201 (4.9%) reported symptoms duration longer than 7 days at the visit.

As shown in Table 1, patients with a median age of 63 years were mostly vaccinated for SARS-CoV-2 (92%) with a median of 3 doses and a low prevalence of comorbidities except for immunosuppressive conditions (median MASS 5/18), among which the most represented were hematologic malignancies (67%). For the whole duration of infection, until the patients were declared virologically cleared, immunosuppressive treatment were modified according to clinical decisions of the reference specialists, shared with infectious disease specialists, without a defined protocol. In all the patients, chemotherapy and monoclonal antibody therapy were withheld until virological clearance, while anti-rejection medication and corticosteroid therapies were continued if not contraindicated for drug interaction with antivirals.

**Table 1.** Demographic and clinical characteristics.

	***N* = 52**
**Age,** years, median (IQR)	63 (50–72)
**Females**, n (%)	28 (53)
**Vaccinated,** n (%)	48 (92)
**SARS-CoV-2 vaccine doses,** median (IQR)	3 (3)
**MASS score,** median (IQR)	5 (3–8)
BMI ≥ 30, n (%)	4 (8)
CKD, n (%)	10 (19)
Diabetes mellitus, n (%)	6 (11.5)
Cardiovascular disease, n (%)	5 (10)
Hypertension, n (%)	16 (31)
Chronic liver disease, n (%)	0 (0)
Chronic respiratory disease, n (%)	6 (11.5)
**Cause of immunesuppression**	
Solid organ transplant recipient, n (%)	8 (15)
Primary immunodeficiency, n (%)	1 (2)
Hypogammaglobulinemia, n (%)	5 (10)
Multiple sclerosis, n (%)	3 (6)
**Hematologic malignancies,** n (%)	35 (67)
*Myeloid malignancies* **,** *n (%)*	4 (8)
*Non-Hodgkin lymphoma* **,** *n (%)*	15 (29)
*Chronic lymphatic leukemia* **,** *n (%)*	3 (6)
*Multiple myeloma/Plasma cellular disorders* **,** *n (%)*	13 (25)
**Other immunodeficiencies,** n (%)	2 (4)
**Use of anti-CD20**, n (%)	13 (25)
**Antiviral combination drug**	
Nirmatrelvir/ritonavir, n (%)	33 (64)
Remdesivir, n (%)	19 (36)
Follow-up, days, median (IQR)	108 (89-192)
**Days from COVID-19 symptoms to combination therapy,** median (IQR)	3 (2-5)
**SARS-CoV-2 infection duration from the end of therapy,** days, median (IQR)	10.5 (6-15.2)
**Reinfection/relapse within 90 days,** n (%)	0 (0)
**Hospitalization within 30 days,** n (%)	0 (0)
**Need for O2 during COVID-19,** n (%)	0 (0)
**Severe ADR,** n (%)	0 (0)
**Deaths within 60 days**, n (%)	0 (0)
**Deaths >60 days**, n (%)	4 (8)

MASS: Monoclonal Antibody Screening Score; BMI: body mass index; CKD: chronic kidney disease; ADR: adverse drug reaction.

After a median of 3 days (IQR 2–5) from COVID-19 symptom onset, the patient started combination treatment (N/r in 64% of cases and remdesivir in 36%). None of the patients were hospitalized within 30 days from symptom onset, needed O2 administration at home, died within 60 days, or experienced a reinfection or a clinical relapse within 90 days. Four patients died 60 days after the beginning of therapy for causes unrelated to COVID-19, of which three were negative for SARS-CoV-2 at NSF, and one died of unknown SARS-CoV-2 status, since he did not attend the follow-up 30 days after therapy administration.

The median duration of SARS-CoV-2 infection from the end of the combination therapy was 10.5 days (IQR 6-15.2) and 17/53 (33%) of the patients had a prolonged infection (≥14 days from the end of the therapy). Nonetheless, nearly all patients (49/51, 97%) reached documented viral clearance at the end of follow-up, except for the abovementioned patient who died 58 days after therapy with unknown SARS-CoV-2 status and another patient who had detectable SARS-CoV-2 at the end of follow-up. The latter patient, a 50-year-old women with chronic lymphoid leukemia treated with venetoclax, had a benign course of COVID-19 and remained with detectable SARS-CoV-2 at the end of follow-up, asymptomatic, with no need for hospitalization or any worsening of her conditions. She reached SARS-CoV-2 clearance in March 2024, 120 days after receiving combination therapy.

As shown in Table 2, patients with prolonged infection were not significantly different with respect to age, sex, vaccination coverage, type of underlying condition, MASS score, and type of combination therapy. Notably, prolonged infection occurred in 11/17 (64%) patients who received antiviral treatment more than three days from symptom onset, compared to 12/35 (34%) patients who received treatment within three days (*p* < 0.05). Moreover, we found no differences in viral clearance between nirmatrelvir/ritonavir and remdesivir. We observed no severe adverse drug reactions, and no patients stopped treatment because of adverse effects.

**Table 2.** Factors associated with viral shedding longer than 14 days. MASS: Monoclonal antibody Screening Score.

	Overall *N* = 52	Viral shedding <14 days *N* = 35	Viral shedding ≥ 14 days *N* = 17	*p*-value
**Age,** years, median (IQR)	63 (50–72)	63 (50–71)	65 (49–74)	0.625
**Females**, n (%)	28 (53)	19 (54)	9 (52)	0.927
**Vaccinated,** n (%)	48 (92)	33 (94)	15 (88)	0.396
**SARS-CoV-2 vaccine doses,** median (IQR)	3 (3)	4 (2-3)	3 (3-4)	0.665
**MASS score,** median (IQR)	5 (3-8)	5 (2-4)	5 (3-6)	0.927
**Solid organ transplant recipient,** n (%)	8 (15)	4 (11)	4 (23)	0.230
**Hematologic malignancies,** n (%)	35 (67)	25 (71)	9 (52)	0.189
**Use of anti-CD20**, n (%)	13 (25)	9 (26)	4 (23)	0.575
**Nirmatrelvir/ritonavir,** n (%)	33 (63)	23 (66)	10 (59)	0.628
**Remdesivir,** n (%)	19 (36)	12 (34)	7 (41)	0.628
**Late treatment (>3 days from symptoms),** n (%)	23 (51)	12 (34)	11 (64)	<0.05
**Treatment started > 180 days from study onset,** n (%)	35 (67)	22 (63)	13 (76)	0.326

**Figure 1.**
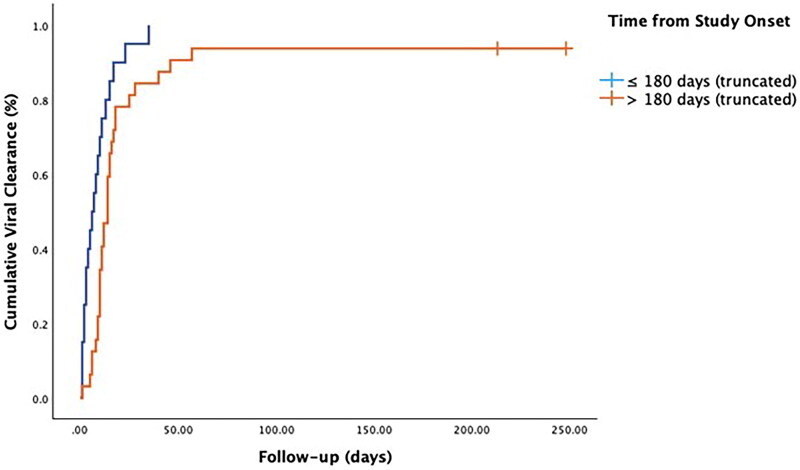
Kaplan-Meier analysis according to time of enrollment from study onset. Log-rank 8,908, *p* < 0.01.

**Figure 2.**
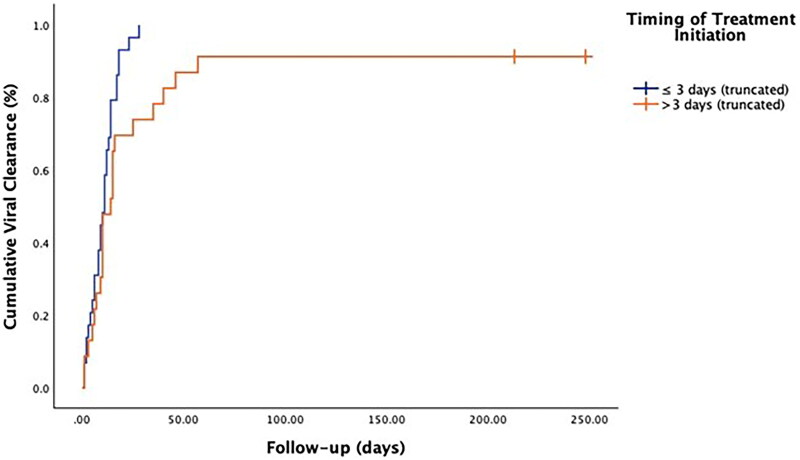
Kaplan-Meier analysis according to timing of treatment initiation. Log-rank 5.095, p<0.05.

Furthermore, we observed a notable variation in the duration of SARS-CoV-2 infection based on the enrollment time from the commencement of the study as well as the time of treatment initiation from the onset of symptoms. Specifically, patients who were enrolled more than 180 days after the study began (corresponding to October 2023) exhibited significantly longer SARS CoV-2 infection (log-rank 8.908, *p* < 0.01) (Table 3, Figure 1). Furthermore, patients who received treatment for SARS-CoV-2 infection later than three days from the onset of symptoms also exhibited significantly longer SARS-CoV-2 infection (log-rank 5.095, *p* < 0.05) (Table 4, Figure 2). In the multivariate Cox regression analysis, enrollment of patients for more than 180 days from the beginning of the study was the only variable independently associated with longer SARS-CoV-2 infection (aHR, 1.94; 94%CI, 1.05-3.56; *p* < 0.05), despite timing of antiviral administration after 3 days retained borderline statistical significance (Table 5).

**Table 3.** Estimated time to SARS-CoV-2 viral clearance depending on time from study onset.

time from study onset (days)	Median			
	Estimate	Standard Error	95% Confidence Interval	
			Lower Limit	Upper Limit
≤ 180 days	6.000	2.236	1.617	10.383
> 180 days	14.000	1.389	11.277	16.732
Overall	11.000	1.078	8.886	13.114

**Table 4.** Estimated time for SARS-CoV-2 viral clearance based on the timing of treatment initiation from the onset of symptoms.

time from study onset (days)	Median			
	Estimate	Standard Error	95% Confidence Interval	
			Lower Limit	Upper Limit
≤ 3 days	11,000	1.326	8.401	13.599
> 3 days	14.000	1.797	10.478	17.522
Overall	11.000	1.078	8.886	13.114

**Table 5.** Cox regression analysis on risk factors for achieving viral clearance later. MASS: Monoclonal antibody Screening Score.

	Univariate Analysis			Multi-variate analysis		
	HR	95%CI	*p*-value	aHR	95%CI	*p*-value
**Age,** years, median (IQR)	0.99	0.95–1.03	0.709	–	–	–
**Male sex**, n (%)	0.77	0.29–2.01	0.619	–	–	–
**Vaccinated,** n (%)	0.37	0.07–1.81	0.220	–	–	–
**SARS-CoV-2 vaccine doses,** median (IQR)	1.60	0.43–5.91	0.483	–	–	–
**MASS score,** median (IQR)	1.04	0.87–1.26	0.631	–	–	–
**Solid organ transplant recipient,** n (%)	3.53	0.97–12.88	0.056	1.063	0.43-2.60	0.893
**Hematologic malignancies,** n (%)	0.39	0.13–1-16	0.091	0.93	0.47-1.84	0.853
**Use of anti-CD20**, n (%)	1.00	0.30–3.28	0.998			
**Nirmatrelvir/ritonavir,** n (%)	0.55	0.20–1.53	0.258	–	–	–
**Remdesivir,** n (%)	1.80	0.65–4.94	0.258	–	–	–
**Treatment started > 180 days from study onset,** n (%)	2.10	1.15–3.83	<0.05	1.94	1.05–3.56	<0.05
**Late treatment (>3 days from symptoms),** n (%)	1.96	1.06–3.63	<0.05	1.85	0.99–3.45	0.054

## 4. Discussion

According to our results, early administration of combination therapy with one direct antiviral agent and the monoclonal antibody sotrovimab in the outpatient setting is associated with high viral clearance, low risk of death, and hospitalization in a cohort of immunocompromised patients with mild-to-moderate COVID-19. None of our patients required hospital admission, oxygen therapy, or died within 60 days of therapy administration for causes related to COVID-19. Moreover, the median duration of SARS-CoV-2 infection in our cohort was 10.5 days (IQR 6-15.2) and only 32% of the patients had viral shedding longer than 14 days from the end of the therapy, with 96% of patients reaching stable virological clearance, with no relapse or reinfections during follow-up.

Persisting viral replication in immunocompromised hosts increases the risk of selecting SARS-Cov-2 variants, which escape from antibody neutralization and mutations which increase antiviral resistance, especially when patients are exposed to multiple therapies in the attempt of reaching virological clearance [10–12]. In the light of this, despite a clear evidence supporting this approach, the combination therapy with one antiviral and a mAb, or two antiviral, with or without a mAb, has become increasingly recommended by experts to treat persistently infected patients, although based on personal opinion or small non-controlled studies [13].

To date, only a few authors have studied the systematic use of combination therapies in immunocompromised subjects, and these studies mostly reported the use of combination therapy in patients who had already developed prolonged or persistent COVID-19, mostly hospitalized[3].

For example, Mikulska et al. reported the use of combination therapy only in hospitalized patients after a median time of 42 (IQR 29–100) days from SARS-CoV-2 infection, with response rates of 75%, 73%, and 82% at day 14, day 30, and last follow-up, respectively [4]. Similarly, D’Abramo et al. recently reported the use of combination therapy in a cohort of 69 immunosuppressed patients hospitalized for severe COVID-19 (92% required oxygen therapy) and treated a median of 21 (IQR 8–36) days from symptom onset [14]. Interestingly, in this study, the use of monoclonal antibodies (tixagevimab/cilgavimab or sotrovimab) in the antiviral combination was associated with a significantly higher rate of viral clearance [14]. In both of the abovementioned studies, the duration of viral shedding was longer than that in our study, but treatment was started later during the course of infection.

On the other hand, a recently published paper by our group analyzed the efficacy and safety of the combination of two antivirals, with or without a mAb, both in early (within 10 days from symptoms) and in the later phase (after 10 days) of SARS-CoV-2 infection in immunocompromised subjects, finding that 100% of the patients treated early reached virological clearance at day 30 from the end of the therapy and were alive and well at follow-up, whereas the corresponding figures in the late-treated patients were 50% and 75%, with patients in the late group more frequently needing oxygen supplementation (*p* = 0.015), steroid therapy (*p* = 0.045), and reaching higher COVID-19 severity (*p* = 0.017) [6].

In line with this, Orth and colleagues have recently presented the largest cohort (144 subjects, of which 82% were immunocompromised) of patients treated with combination therapy [5], according to co-primary endpoints (prolonged viral shedding at day 21 after treatment initiation and days with SARS-CoV-2 viral load ≥ 10^6^ copies/ml). The authors found that underlying hematological malignancies and treatment initiation later than five days after diagnosis were significantly associated with longer viral shedding, which [5] was confirmed and consolidated by our results, since we found a significantly higher proportion of patients with prolonged infection (64%) among those who started antiviral therapy later than 3 days after symptoms (*p* < 0.05). The delay in administering the combination therapy was significantly associated with a longer SARS-CoV-2 infection duration (HR 1.96; 95%CI 1.06–3.63, *p* < 0.05), although this result was not confirmed by multi-variate analysis.

In the largest part of published studies which included a safety analysis of the combination approach, the reported adverse events were mostly mild and not requiring drug discontinuation. The most commonly reported adverse events were sinus bradycardia, which is already described with remdesivir and that led to remdesivir discontinuation in the cohort of Mikulska and Gentile [4,6].

Interestingly, we observed that the independent risk factor for not achieving early SARS-CoV-2 clearance in our study was the enrollment in the last months of the study (from October to the end of December. We speculate that this could be related to the loss of efficacy of sotrovimab against new circulating variants of SARS-CoV-2. In fact, during the study period, there has been a shift from a prevalent circulation of the XBB1.5 variant, against which sotrovimab retained *in vitro* efficacy, towards new variants (XB1.9, BA2.86, and JN1), against which sotrovimab showed higher 50% inhibitory concentrations (IC50) and therefore potentially lower neutralizing activity [15,16]. In fact, binding and viral neutralization efficacy of sotrovimab were recently found to be totally abolished by the emergence of the 2023 FLip’s lineages and in BA.2.86 carrying the Spike mutation K356T [17,18]. This consideration is underpowered by the fact that we did not assess the SARS-CoV-2 strains by which the patients were infected, and we did not test the neutralization capacity of sotrovimab in each case.

Another important limitation of the study is the lack of a control group of patients with similar immunosuppression, but treated with a single therapy, to assess the real advantage of a combination of antivirals and sotrovimab compared to antivirals alone. By comparing our results to historical cohorts, we found a great reduction in mortality and needing for mechanical ventilation, when we consider observational studies with low prevalence of early antiviral or mAb use [19], but also a low prevalence of hospital admission (4%) and persistent infection (1.6%) in immunocompromised patients who receive early treatment with N/r alone [20]. Recently, Mazzitelli et al. have published a retrospective study comparing 30-day mortality, access to emergency department and hospitalization between immuncompromised COVID-19 patients treated with antivirals alone and antivirals plus sotrovimab [21]. They found that no significant differences were observed between the two groups for the outcomes taken individually, but, after applying a propensity score weighted approach, they found that combination therapy, and both altered liver and kidney function, were significantly associated with the composite outcome, in a favourable and unfavourable manner, respectively [21].

These contrasting findings need to be further analyzed with new studies specifically aimed to comparing monotherapy versus combination therapy in immunocompromised patients. Finally, we emphasize that in our study, we used two different direct antivirals with different mechanisms of action, together with sotrovimab. However, we did not find any difference in the main outcomes between the two different antivirals employed.

## 5. Conclusions

Despite its limitations, our study suggests that early administration of combination therapy with sotrovimab and a direct antiviral agent is safe and could be effective in preventing hospitalization, progression to severe COVID-19, and the development of prolonged/persisting SARS-CoV-2 infection in severely immunocompromised patients. The circulation of new variants could prevent the efficacy of this strategy due to the loss of efficacy of sotrovimab. Further studies are required to compare the combination approach with monotherapy in these categories, especially considering the reduced activity of the monoclonal compound.

## Author’s contributions

IG, ARB, and GV conceptualization; RS methodology and software; FC, DP, ADA, MS, SM, FG, SP, AG, FT, MM resources, formal analysis, investigation; GV, ARB, RS writing original draft; FT, IG, MM Writing - Review & Editing. All authors have read and approved the manuscript.

## Ethics approval and consent to participate

All procedures performed in this study were in accordance with the ethical standards of the institutional and/or national research committee and with the 1964 Helsinki declaration and its later amendments or comparable ethical standards. This study was approved by the Ethical Committee of the University of Naples Federico II (protocol no. 98/22).

## Consent for publication

Written patient consent was obtained prior enrollment to in the study, and the data were recorded by the investigators anonymously such that subjects could not be identified directly or through identifiers linked to the subject.

## Data Availability

The data supporting the findings of this study are available from the corresponding author upon reasonable request. The manuscript has been published in pre-print on July 15, 2024, and available at: http://medrxiv.org/content/early/2024/07/15/2024.07.15.24310384.abstract
